# Supplementary material for: Comprehensive Annotation and Functional Exploration of MicroRNAs in Lettuce
Source: Front Plant Sci. 2021 Dec 24;12:781836. doi: 10.3389/fpls.2021.781836 (PMC8739914; doi:10.3389/fpls.2021.781836)
Supplement: Supplementary file 3 [file Data_Sheet_9.ZIP › GSM2230753.plot/Lsa-miR396e_Lsat_1_v5_gn_4_119461.1_113_TPlot.pdf]

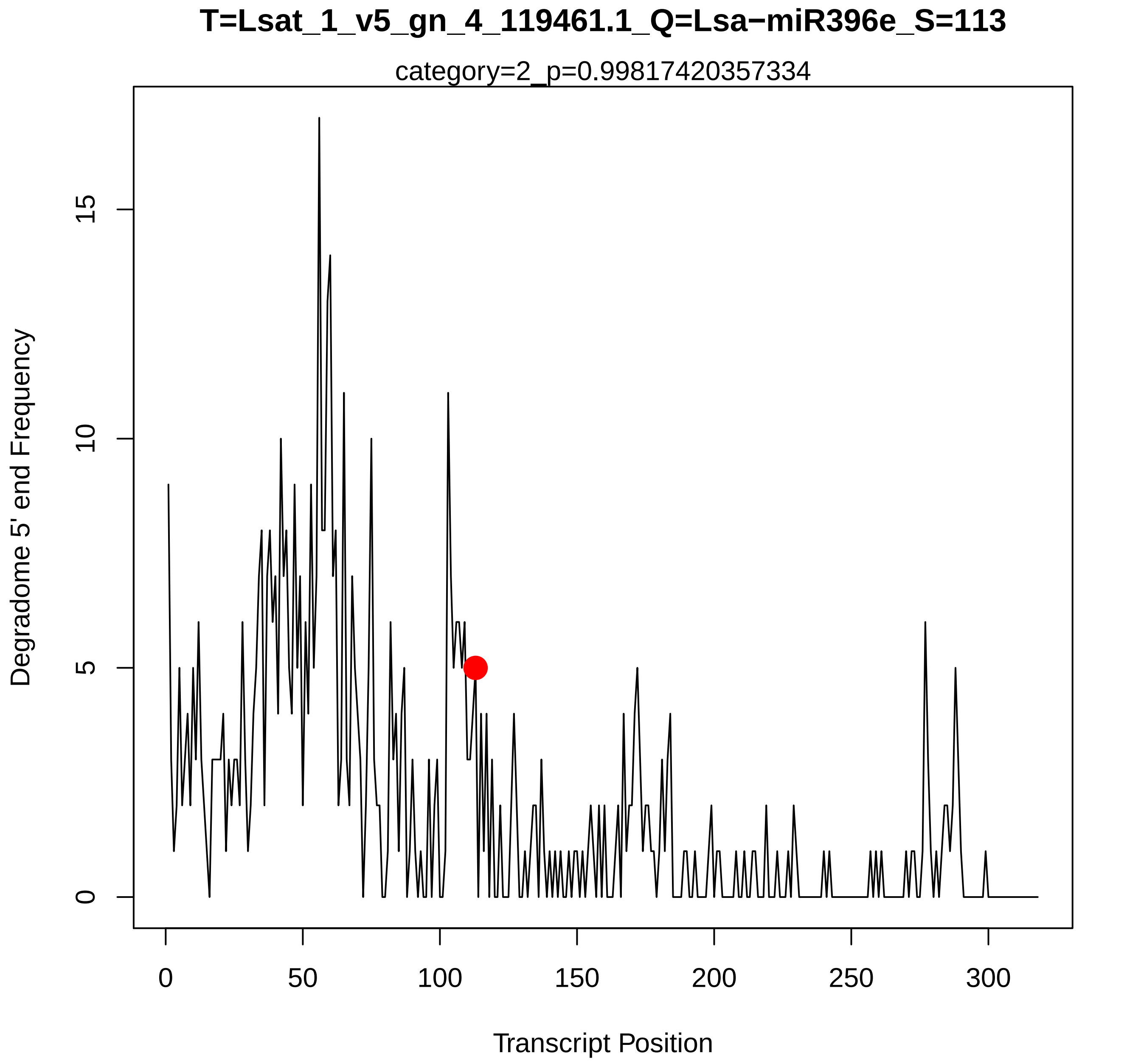

T=Lsat_1_v5_gn_4_119461.1_Q=Lsa−miR396e_S=113
category=2_p=0.998174203573334
Degradome 5' end Frequency
0
5
10
15
0
50
100
150
200
250
300
Transcript Position
